# Increasing the options for reducing adverse events: Results from a modified Delphi technique

**DOI:** 10.1186/1743-8462-5-25

**Published:** 2008-11-14

**Authors:** Jeff Richardson, John McKie

**Affiliations:** 1 Centre for Health Economics, Faculty of Business and Economics, Monash University, Clayton, Victoria 3800, Australia

## Abstract

**Background:**

The aim of this paper is to illustrate a simple method for increasing the range of possible options for reducing adverse events in Australian hospitals, which could have been, but was not, adopted in the wake of the landmark 1995 'Quality in Australian Health Care' study, and to report the suggestions and the estimated lapse time before they would impact upon mortality and morbidity.

**Method:**

The study used a modified Delphi technique that first elicited options for reducing adverse events from an invited panel selected on the basis of their knowledge of the area of adverse events and quality assurance. Initial suggestions were collated and returned to them for re-consideration and comment.

**Results:**

Completed responses from both stages were obtained from 20 of those initially approached. Forty-one options for reducing AEs were identified with an average lapse time of 3.5 years. Hospital regulation had the least delay (2.4 years) and out of hospital information the greatest (6.4 years).

**Conclusion:**

Following identification of the magnitude of the problem of adverse events in the 'Quality in Australian Health Care' study a more rapid and broad ranging response was possible than occurred. Apparently viable options for reducing adverse events and associated mortality and morbidity remain unexploited.

## Background

Results from the 1995 'Quality in Australian Health Care' (QAHC) study suggested that the quality of health care in Australia is a problem that overshadows all others in the health sector. In the initial study, reported by Wilson et al. [1], medical records for more than 14,000 admissions to 28 hospitals in NSW and SA in 1992 were individually examined to determine whether or not an adverse event (AE) was associated with the admission (prior to or during the episode of hospitalisation). A team of medical officers then made a judgment concerning the degree of preventability of the AE.

By extrapolating results the authors estimated that about 470,000 admissions were associated annually with an AE and that these would have resulted in 18,000 deaths and 50,000 cases of permanent disability. In a subsequent report, Runciman et al. [2] estimated that 50% of the AEs in the QAHC study had a high preventability score. Sixty per cent of deaths could have been avoided. In this latter study, incidence and not prevalence scores were reported as part of the effort to standardise the methodology with an earlier Harvard Medical Practice Study (HMPS) reported by Brennen et al. [3]. This reduced the annual rate of AEs to 10.6% of admissions.

The direct hospital costs of adverse events, both fatal and non-fatal, were estimated in the QAHC study at A $900 million per annum. This was likely to have been conservative 'as the costs of such problems arising in mental health, nursing homes, domiciliary care, day patients, and general or specialist practice outside such hospitals were not included' [[4], p. 7]. Moreover, the cost was based on each hospital day attributed to an adverse event costing the same as the average of all hospital days, whereas the evidence from other studies suggests that hospital stays associated with adverse events cost more than average. Using data from the study, Rigby et al. estimated that the cost of treating 12 conditions, representing just 22% of the adverse event categories, was A $483 million [[4], p. 9].

Subsequent studies have confirmed the existence of a major problem. For example, using Victorian Department of Human Services data, representing 90% of the direct hospital expenditure in Victoria, Ehsani et al. found that 6.88% of routine admissions were associated with a coded adverse event [5]. The discrepancy in the incidence of the problem reported in the latter study, and that reported in the original QAHC study, is undoubtedly attributable in part to the methodology. In principle, the dedicated Wilson et al. (1995) method of screening, and the individual, multi-speciality examination of each record, should identify a larger number of AEs than the routine classification of records by hospital staff. Ehsani et al. also confirmed the financial consequences of AEs. According to their calculations, separations associated with an AE had an additional cost of $2 billion nationally per annum, or an additional 18.6% of the total inpatient hospital budget. If 50% of these were preventable (the figure used by Runciman et al. based on the QAHC data) this would represent $1 billion nationally.

Following the QAHC study a taskforce under the direction of Bruce Armstrong was established to look into the problem. Its report was shelved after a challenge to the validity of the QAHC study. This resulted in a study by Runciman et al. [2], the creation of an expert 'group' of four and, eventually, the Australian Council for Safety and Quality in Health Care (ACSQHC) in 2000 [6]. This in turn has been replaced by the Australian Commission on Safety and Quality in Health Care. The activities of ACSQHC are summarised in a number of annual reports. In addition, the Australian Health Care agreement between the Commonwealth and State governments allocated budgets of $680 million and $785 million for quality assurance activities for the periods 1998–2003 and 2003–2008. In each of the States, sub-committees and working groups were created which, along with the ACSQHC, have resulted in a large number of reports, publications, some legislation and local initiatives. State activities are summarised in the ACSQHC's *Safety Innovations in Practice (SIIP) Program Mark II, Compendium of Project Reports *[7]. In 2004 the Clinical Excellence Commission (CEC) was launched, as part of the New South Wales Patient Safety and Clinical Quality Program. It's mission is 'to build confidence in healthcare in NSW, by making it demonstrably better and safer for patients and a more rewarding workplace' [8].

Despite this level of activity, the response to what might justifiably be described as a crisis in Australian hospitals has been cautious and incremental. Presumably the results of the QAHC study were known for some time before publication as the admissions data used in the study was from 1992. It is scarcely surprising that in 1999 an editorial in the *Medical Journal of Australia *commented that, although the various initiatives are welcome, 'the pace of change nevertheless seems slow given the stark message of the original QAHC study four years ago.... 50,000 Australians suffer permanent disability and 18,000 die at least in part as a result of their health care' [9]. By 2002 Siddons could still comment that, 'On the 10th anniversary of the study year, the most striking outcome has been the paucity of reform currently exhibited at the coalface of tertiary health care' [[10], p. 823] and by 2005 an MJA editorial could still question whether or not any improvement had been achieved in the previous 10 years [11].

While the exact dimensions of the problem were debated there was no suggestion at any stage that Australia did not face a very serious problem. One of the themes of the present paper is that the response to this could have, and should have, been significantly faster and more effective. Some of the problems responsible for AEs addressed below were self-evident, and immediate administrative and possibly legislative action might have been justified, albeit with close monitoring and review following confirmation of the causal factors: with preventable deaths reportedly occurring at a rate equivalent to a Bali bombing every 3 days, haste was justified but did not occur.

One simple methodology demonstrating how this might have been achieved is described below. We report the results of a survey conducted among professionals diversely associated with the health industry that sought to canvass practical measures for addressing the problem of adverse events in hospitals. The purpose was not to create a comprehensive check-list of possible interventions but, rather, to demonstrate the feasibility of the approach, and act as a conduit for channelling options to policy makers and legislators. Some of these have subsequently been adopted into policy but we have made no attempt to screen these out as they indicate advice that was immediately available but often not acted upon. Other potentially important measures have still not been adopted. A second reason for the study is the belief that the identification of even modest new options, or the circulation of proposals currently under discussion, along with expert opinion about their feasibility, may be of value due to the size of the problem being addressed and the magnitude of benefits from even incremental improvements. The approach does not purport to be a definitive or an authoritative solution to the problem of AEs. It is more akin to a 'brainstorming' which seeks to throw up divergent ideas, some more feasible than others, to enlarge the scope of ideas for consideration.

## Method

We consulted a number of Australian experts in health safety and quality issues. Their names were obtained from public domain resources: authors of published articles on safety and quality issues, departmental officials in the area of safety, those on relevant committees, conference participants, and AE researchers known to the investigators. Of the 76 individuals who were sent invitations, 23 held positions in quality control or practice improvement (19 of these were Managers, Directors, Chairmen or Co-ordinators). The next largest group comprised 16 experts in clinical governance/management or clinical risk management (13 of these were Managers, Heads, Co-ordinators or (Acting/Deputy) Directors). There was some overlap between these two groups, e.g. when clinicians were Managers of quality control units.) Invitations were also sent to representatives of government departments (10), to senior nurses (5) and academics (5). The rest were made up of epidemiologists, business people, and 9 individuals for whom we had an affiliation (e.g. hospital or university) but no position description. Invitees were asked to make their suggestions anonymously.

The study adopted a modified Delphi methodology using a two-stage procedure. First, we sent a questionnaire describing a number of proposals for improving safety and quality and asked recipients to comment on the options and to make additional suggestions. The questionnaire was divided into 7 sections. These were: (1) error learning, (2) hospital accreditation, (3) hospital information systems, (4) out of hospital information, (5) other hospital regulation, (6) doctors, and (7) system level reform. New proposals from the first round were added to the original list and returned to the experts for comment on their feasibility and potential impact. Specifically, they were asked in the second stage to rate on a six-point scale: (i) the potential effect of each proposal (very high, high, low, very low, none, negative), (ii) how quickly they believe it could be implemented (immediate, one month, six months, one year, five years, ten years (or more)), and (iii) the time before the option would be likely to have a major impact (immediate, one month, six months, one year, five years, ten years (or more)). They were also asked to write comments on the proposals, including arguments for and against their adoption.

Terminology in this area is not uniform. In articulating the proposals we adopted the preferred terms and definitions used by the ACSQHC [12]. In particular, the following definitions were given along with the proposals. 'Adverse Event': An incident in which harm resulted to a person receiving health care. 'Incident': An event or circumstance which could have, or did lead to unintended and/or unnecessary harm to a person, and/or complaint, loss or damage. 'Harm': Death, disease, injury, suffering, and/or disability experienced by a person. 'Health Care': Services provided to individuals or communities to promote, maintain, monitor, or restore health. Health care is not limited to medical care and includes self-care.

The analysis was essentially qualitative, not quantitative, and hypothesis testing of the results is therefore not appropriate. The objective was to demonstrate the methodology, elicit potentially good ideas, and determine their feasibility according to the prevailing views of a group of experts. A single idea from a single person might be more fruitful than the shared beliefs of a majority. For this reason also, survey response rates, detailed respondent characteristics and representativeness of respondents are of little interest for the main purposes of the study.

## Results

Of the 76 individuals to whom invitations were sent, completed results from both stages of the survey were obtained from 20. For the reasons noted above and discussed later this relatively low response rate does not invalidate the results or subtract from the potential value of the suggestions made.

### (1) Error Learning and Mandatory Disclosure

The first section dealt with error learning and mandatory disclosure. The proposals in this section were rated close to 'high' in terms of their potential effect. The highest score reported in Table 1 was associated with the collection and reporting of information on preventable post-discharge complications after elective surgery (P 1.9). There was also support for the mandatory reporting of adverse events (P 1.2), for the mandating of procedures that would facilitate and encourage the reporting of incidents by patients, (P 1.4), and for mandatory incident reporting and mandatory review of systems following an unexpected increase in the frequency of incidents (P 1.5). The degree of support among experts for mandatory measures has not been systematically investigated, but the results reported here suggest that it may be strong. Those consulted thought it would take less than a year to implement the proposals dealing with compulsory disclosure and less than another year before they would have a major effect.

**Table 1:** Error learning and mandatory disclosure

**Proposals**	**Effect^a ^**[1 = v.high 6 = negative] Mean (std dev)	**Implement^b ^**(months)	**Impact^c ^**(months)	**Total^d ^**(years)
**1.1 **Providers, including doctors and hospitals, should receive immunity against litigation relating to adverse events that have been reported, and compensation to an injured party or parties should be paid for from a subsidised government or privately run claims-fund where compensation is not contingent upon blame.	2.17 (.79)	42	55	8.38
**1.2 **Reporting of adverse events should be mandatory.	2.29 (1.45)	9	9	1.5
**1.3 **Remedial or punitive action against service providers should be independent of compensation paid, and all providers should be affiliated with an accredited body which reviews, collates and provides summary information about adverse events to providers.	2.35 (1.00)	26	26	4.3
**1.4 **Procedures should be mandated that facilitate and encourage the reporting of incidents by patients.	2.19 (1.11)	9	8	1.4
**1.5 **There should be mandatory incident reporting not just mandatory disclosure of adverse events, and mandatory review of systems following an unexpected increase in the frequency of incidents, in all public and private hospitals.^e^	2.18 (1.29)	11	9	1.7
**1.6 **There should be complete reporting of all patient outcomes, not only incident reporting.	2.41 (1.66)	64	28	7.7
**1.7 **All hospital deaths should be reviewed by an independent clinical governance department and deaths suspected of being related to an adverse event, plus any action take to prevent such events in the future, should be reported.^f^	2.31 (1.08)	10	18	2.3
**1.8 **All implanted devices, such as pace makers, should have a unique identifier. Whenever a clinician or patient reports a problem, or when a device is removed, or a patient dies with a device in place, this should be recorded on an electronic data base. This data base should be continually monitored for patterns that might indicate a problem.^g^	2.33 (.97)	10	21	2.6
**1.9 **Hospitals should collect and report data on preventable post-discharge complications after elective surgery.	1.93 (.59)	11	21	2.7

a Respondents were asked to rate the potential effect of the option on a six-point scale: 1 = very high, 2 = high, 3 = low, 4 = very low, 5 = none, 6 = negative. Lower scores indicate a higher potential effect.

b How quickly it could be implemented. First the mean was calculated using the following scale: 1 = immediate, 2 = one month, 3 = six months, 4 = one year, 5 = five years, 6 = ten years (or more). Then the result was converted into months. For example, a mean score of 4.63 = 1 yr + 63% of 48 months (5 yrs – 1 yr) = 42 months (1 yr + 30.24 months).

c Time before the option would be likely to have a major effect: 1 = immediate, 2 = one month, 3 = six months, 4 = one year, 5 = five years, 6 = ten years (or more). See above for method of converting into months.

d Implementation time plus impact time.

e In Victoria mechanisms are currently in place to ensure that Root Cause Analysis (RCA) is conducted following an increase in the frequency of incidents in public hospitals.

f The Quality and Safety Branch of the Victorian Department of Human Services and the State Coroner's Office already carry out these functions in Victoria with full investigation and recommendations are promulgated.

g To some extent this already occurs – e.g. in the case of heart valves [31] and joint replacements [32].

### (2) Hospital Accreditation and Audit

Responses to proposals concerning the hospital accreditation and auditing process are summarized in Table 2. Undertaking regular anonymous surveys of medical and nursing staff for feedback on the safety and quality climate in the hospital was rated high in terms of its potential effect, to be capable of implementation within six months, and was thought likely to have a major impact within a year. Confirming the support for mandatory procedures evident in the last section, there was support for compulsory accreditation (P 2.1) and for the mandatory auditing of identified high-risk hospitals (P 2.6).

**Table 2:** Hospital accreditation and audit

**Proposals**	**Effect **[1 = v.high 6 = negative] mean (std dev)	**Implement **(months)	**Impact **(months)	**Total **(years)
**2.1 **Accreditation should be compulsory for all public and private hospitals and day surgery facilities.	2.16 (.83)	9	26	2.9
**2.2 **There should be a review of the criteria for achieving accreditation. Criteria should be expanded to include more stringent procedures relating to safety.^a^	2.17 (.99)	18	30	4.0
**2.3 **Accreditation should be more focused on measurable outcomes which should be standardised to allow benchmarking against other hospitals.	2.65 (1.06)	12	41	4.4
**2.4 **Hospitals should undertake regular anonymous surveys of medical and nursing staff to get feedback on the safety and quality climate in the hospital.^b^	2.22 (.94)	6	11	1.4
**2.5 **There should be no forewarning of the date on which the accreditation review occurs. Accreditation should include follow-up reviews on random dates. All hospitals should be subject to random audit of facilities and procedures.	2.89 (1.66)	9	12	1.8
**2.6 **The audit procedures used in the Quality in Australian Health Care Study^c ^should be introduced as a permanent feature of the public and private hospital systems, with mandatory auditing of identified high-risk hospitals, and random auditing of the remainder.	2.33 (.97)	12	24	3.0
**2.7 **The criteria for accreditation should be subject to evaluation against known standards for the achievement of safety and quality. Where possible, the results of random control trials should be the basis for the inclusion or rejection of criteria and standards.	2.33 (.84)	26	42	4.8

a The Australian Health Ministers have recently endorsed the release of a Discussion Paper on National Safety and Quality Accreditation Standards as the basis for consultation with stakeholders [33].

b We note that some hospitals already do this – e.g. the Royal Children's Hospital in Victoria.

c Wilson, R. M., W. B. Runciman, R. W. Gibberd, B. T. Harrison, L. Newby and J. D. Hamilton (1995). 'The Quality in Australian Health Care Study.' *Medical Journal of Australia *163(9): 458–471.

### (3) Hospital Information Systems

The third section, summarized in Table 3, contained four proposals dealing with in-hospital information systems. The idea of tailoring clinical pathways to individual patients (P 3.2) received less support in terms of its potential effect than the other proposals, and was judged to require a longer period before a major effect would be felt, possibly because the requirement that pathways be based on 'full information regarding patient history and co-morbidities' was thought to impose an excessive burden on clinical staff. In general, however, the four proposals dealing with in-hospital information systems were rated high in terms of their potential effect, albeit with varying implementation and impact times.

**Table 3:** Hospital information systems

**Proposals**	**Effect **[1 = v.high 6 = negative] mean (std dev)	**Implement **(months)	**Impact **(months)	**Total **(years)
**3.1 **As a condition of accreditation all hospitals meeting designated criteria with respect to patient numbers and case complexity should have an appropriate internal information system for recording patient history, treatment (including drugs), digitized radiological imaging, pre- and post-discharge requirements.	2.11 (.68)	33	26	4.9
**3.2 **Clinical pathways should be tailored to individual patients and based on full information regarding patient history and co-morbidities, and not be geared to the average patient.^a^	2.71 (1.10)	30	35	5.4
**3.3 **All medical handovers should be documented in writing to minimise errors due to lack of continuity of care.	2.39 (1.14)	8	10	1.5
**3.4 **All hospitals should have systems in place to identify patients who become acutely ill and to summon appropriate expertise to the bedside within minutes.	2.00 (.89)	8	12	1.7

a The Victorian Department of Human Services has pointed out that 'clinical pathways', by their nature, are geared to the average patient, which ensures that core sets of tools are utilised. However, they agree that 'the clinical pathway should allow for variances based on clinical judgement and patients within a known Diagnosis Related Group (DRG)'.

### (4) Out of Hospital Information

Among other things, Table 4 reveals the potential effect of a non-compulsory 'smart card' (P 4.1). This card would allow aspects of a patient's medical history to be accessed by health care providers at the patient's discretion. Voluntary ownership of such a card, and control over the amount and type of information disclosed, eliminates some of the privacy concerns associated with such measures, and the proposal received a 'high' rating from our respondents in terms of its potential effect. Like the other proposals in this section, our panel believed it would take several years to implement, but, unlike the other proposals, it would have a major impact in less than a year once implemented. By contrast, the positive effect of making information on comparative risk adjusted mortality and adverse event rates available to the public (P 4.2) or Private Health Insurance Funds (P 4.3) received the lowest effect ratings in the survey – both 'Low'. This may reflect the tendency to deal with safety and quality problems 'in-house' rather than expose doctors and hospitals to criticism from without. Alternatively – and as suggested by more than one of our respondents – such information might be difficult for private insurance organizations to interpret and measure.

**Table 4:** Out of hospital information

**Proposals**	**Effect **[1 = v.high 6 = negative] mean (std dev)	**Implement **(months)	**Impact **(months)	**Total **(years)
**4.1 **All Australians registered with Medicare should be offered the option of a smart card which contains the patient's full medical history, and/or the option of having their medical history kept on a centralized database. As an option, the card or database should have a 'secure' record of personal information which the patient wishes to keep confidential under normal conditions and which can be transferred from the patient to the doctor using a confidential PIN. All health care providers should have (subsidized) facilities for accessing information which is not confidential.	2.00 (.71)	47	9	4.7
**4.2 **Comparative risk adjusted mortality and adverse event rates should be on the provider website and freely available to the public. Providers should be allowed to comment on these data when the comment is informational and not marketing for the practice. The date by which this information must be posted should be determined by the volume of procedures and the elapsed time until the numbers allow the information to be statistically reliable. In the interim, process information should be provided.	3.06 (1.14)	43	52	7.9
**4.3 **The government should provide summary hospital data to Private Health Insurance Funds. Funds should be encouraged to use this data when contracting with hospitals.	3.27 (1.10)	33	50	6.9
**4.4 **Each State and Territory Health Department should routinely link discharge and re-admission data to determine the likelihood of an incident-related re-admission within a defined period. This provision should, subsequently, be extended to include data from the Health Insurance Commission and individual-level mortality statistics. Criteria should be developed to identify hospitals, hospital teams, and individual practitioners with an atypically high level of adverse events, to report on between-hospital variation, and to identify areas for improvement.^a^	2.78 (1.17)	40	30	5.8
**4.5 **As in parts of the United States and the United Kingdom, information should be available to the public, including on the internet, regarding risk-adjusted mortality and adverse event by cause for all public and private hospitals. Data should only be posted where the number of cases is sufficiently large that a statistically significant pattern could be expected to emerge. When case loads are below this threshold, this fact and other process information should be made available. Independent groups (such as consumer organisations) should be funded to interpret and disseminate this information. (This later step is needed or, as in some US states, there will be minimal impact of information.)	2.94 (1.14)	35	43	6.5

a It should be noted that re-admission is not always related to an adverse event, and therefore is not a reliable indicator on its own.

### (5) Other Hospital Regulation

Section five dealt with other hospital regulation. (See Table 5.) In terms of its potential effect, the proposal that new or extant procedures should be formalised to guarantee anonymity and/or protection for whistle blowers (P 5.2) was rated third among all proposals, casting doubt on the suggestion above that respondents believe safety and quality issues problems should be dealt with 'in-house'. Also receiving strong support was the idea that hospital staff should assume 'ownership' of safety and quality issues (P 5.4), and that this can be encouraged by training staff in risk management.

**Table 5:** Other hospital regulation

**Proposals**	**Effect **[1 = v.high 6 = negative] mean (std dev)	**Implement **(months)	**Impact **(months)	**Total **(years)
**5.1 **Regulation should require a defined minimum complement of qualified staff in situ (or in close proximity) following defined procedures in all public and private hospitals, where the required minimum is determined by patient safety during the high-risk period of recuperation.	2.00 (.79)	12	9	1.8
**5.2 **All hospitals should have in place a risk management system that ensures personnel can initiate action to prevent and/or reduce the impact of risks. Whistle-blower procedures should be formalised to guarantee anonymity and/or protection for whistle blowers.	1.82 (.73)	8	10	1.5
**5.3 **All hospitals should have trained, specialized risk management staff.	2.24 (.75)	9	11	1.7
**5.4 **All hospital staff should be trained in risk management, so that all staff assume 'ownership' of safety and quality issues.^a^	2.00 (.94)	12	11	1.9
**5.5 **All hospitals should have in place an equipment replacement program and dedicated funding should be made available annually to replace unsafe equipment. This funding should not be part of the overall budget.	2.19 (.91)	11	31	3.5
**5.6 **All university hospitals should have medical education departments for (a) education, (b) credentialing and (c) simulation.	1.94 (.57)	31	19	4.2

a There is some scope for disagreement about what this might mean in practice. For example, the Victorian Department of Human Services believes 'that all staff should be aware of RCA processes (Root Cause Analysis) but need not be fully trained in conducting a RCA'.

### (6) Doctors

Responses to the suggestions directly effecting doctors are reported in Table 6. Three of the proposals in this section were rated in the top five overall in terms of their potential effect. The proposal that the supervision and support of junior doctors should be improved (P 6.4) was rated highest overall. This was also judged to be quickly implementable – within nine months – and would be likely to have a major impact upon AEs within another seven months. Also rated high was the potential effect of credentialing medical students who become interns before conducting unsupervised procedures (e.g. inserting nasogastric tubes). There was also support for centres of excellence that are dedicated to certain procedures – e.g. colon cancer surgery – when it is known that the outcome of such procedures is influenced by the quality of the practice setting or the case load of the unit or doctor (P 6.2). The establishment of such centres would of course be a long-term goal, thus explaining the longer estimated implementation and impact times.

**Table 6:** Doctors

**Proposals**	**Effect **[1 = v.high 6 = negative] mean (std dev)	**Implement **(months)	**Impact **(months)	**Total **(years)
**6.1 **Criteria should be mandated to determine a doctor's right to perform some procedures. These should require periodic review of death rates and adverse events (inter alia). When adverse events and mortality are associated with an attribute of a practice that is known to increase risk (such as small numbers of patients, service delivery to inappropriate patients, or where clinical indicators suggest the procedure is unwarranted) review might be followed initially by advice to alter the unsafe practice or procedure and subsequently, if appropriate, by disaccreditation for that procedure.	2.00 (.73)	22	22	3.7
**6.2 **Centres of excellence should be established that are dedicated to certain procedures – e.g. colon cancer surgery – when it is known that the outcome of such procedures is influenced by the quality of the practice setting or the case load of the unit or doctor.	1.81 (.75)	45	18	5.3
**6.3 **All medical students who become interns should be 'credentialed' before they are allowed to do any unsupervised procedures (e.g. inserting nasogastric tubes).	1.94 (.77)	9	9	1.5
**6.4 **The supervision and support of junior doctors should be improved.	1.56 (.63)	9	7	1.3
**6.5 **All new graduates and all new entrants into the system (e.g. foreign graduates) should have regular performance reviews by medical educators – say, every three months.	2.20 (.68)	9	11	1.7
**6.6 **All hospital doctors should provide e-mail addresses so that hospitals can communicate new protocols, safety rules, etc.	2.33 (.82)	7	10	1.4

### (7) System Level Reform

The final section of the survey dealt with system level reforms. (See Table 7.) Not surprisingly, it was thought these options, in general, would take longer to implement, and that their effect would take longer to be felt. Among proposals aimed at system level reform, the idea that higher payments should be made for practices that are known to improve safety – e.g. the use of approved protocols (P 7.1) – rated highest in terms of potential effect. This idea has been adopted in the UK, where GPs have obtained a significant pay rise for complying with safety and quality indicators.

**Table 7:** System level reform

**Proposals**	**Effect **[1 = v.high 6 = negative] mean (std dev)	**Implement **(months)	**Impact **(months)	**Total **(years)
**7.1 **Higher payments should be made throughout the public and private system for practices that are known to improve safety. Private insurance companies should be mandated to comply with this regulation. Practices known to improve safety might include (a) the use of approved protocols, (b) the performance of procedures in a hospital or facility specifically accredited for the procedure, (c) conduct of the procedure by a specifically accredited provider (several accreditation categories may be desirable).	2.13 (.92)	24	31	4.6
**7.2 **There should be independent analysis at the national level, as well as individual hospital analysis, of adverse events, to assist in the identification of rare but catastrophic events.	2.50 (.86)	11	27	3.2
**7.3 **A National Benchmarking Centre for Clinical and Public Health Outcomes should be established to provide hospitals and clinical managers with ready access to standardised outcomes measures for all treatments, particularly major adverse-event causing treatments.	2.41 (.80)	35	32	5.6
**7.4 **A National Centre for The Development of Clinical Guidelines and Clinical Pathways should be established to (a) promote evidence-based practice, (b) fund, support and disseminate evidence-based clinical guidelines, and (c) prepare model clinical pathways to assist hospitals plan and organise care.	2.27 (.88)	26	31	4.8

## Discussion

The purpose of this survey was twofold. First, it sought to demonstrate that a method was available – and remains available – for identifying reform options that may be fairly rapidly implemented if subsequent inquiry endorses their feasibility. Implicit in this suggestion is the view that it is not necessary or appropriate when people are dying in large numbers to follow exhaustive, time-consuming processes employing existing and conventional channels and designated authorities. Secondly, it sought to demonstrate this by identifying options that might have been – and in most cases still may be – considered for reducing AEs. We discuss these aims in reverse order.

### Options and Timelines

In contrast with the view that little can be done quickly, a number of suggestions were raised in the present study which, according to our panel, could effect significant and effective change fairly rapidly. In Table 8, which summarises the results, the average lapse time of the 41 interventions before having a significant impact was 3.5 years. The 19 options expected to have an impact within 3 years had an average lapse time of 1.8 years before having a significant effect. Measures affecting doctors directly and the regulation of hospitals had a particularly small delay (2.5 years and 2.4 years respectively). At the other extreme, out of hospital information was felt to have a slow effect.

**Table 8:** Summary of lapse time before significant effect

**Category**	**Proposals (N)**		**Average lapse time (years)**	**Category**	**Proposals (N)**		**Average lapse time (years)**
					
	**Impact < 3 years**	**Total in this category**			**Impact < 3 years**	**Total in this category**	
1. Error Learning and Mandatory Disclosure	6	9	3.6	5. Other Hospital Regulations	4	6	2.4
2. Hospital Accreditation and Audit	3	7	3.2	6. Doctors	4	6	2.5
3. Hospital Information Systems	2	4	3.4	7. System Level Reform	0	4	3.6
4. Out of Hospital Information	0	5	6.4	Total	0	4	3.5

A distinguishing feature of some of the suggestions is that they involve regulatory enforcement which appears to be inconsistent with the apparent emphasis upon persuasion and voluntary culture-change evident in many of the initiatives suggested in official reports. In some cases, mandated options gained support in preference to the approach that 'treats the health provider as if it exists in isolation from its environment, oblivious to the institutional, social and economic pressures that drive organisational willingness to contemplate internal reforms' [[13], p. S56].

Healy and Braithwaite articulate a 'pyramid' of regulatory mechanisms with 'soft' options at the bottom (personal monitoring, continuing education...), which progresses through increasingly more stringent regulatory measures (peer review, published performance indicators, external clinical audit...), up to 'command and control' at the top (criminal or civil penalty, licence revocation or suspension...). The pyramid consists of 27 'mechanisms', 14 of which fall within the general categories of 'voluntarism', 'market mechanisms' or 'self-regulation'. It is doubtful that these mechanism alone will have the desired effect, but rather that, for example, 'dependence on voluntary reporting systems will lead to a gross and inconsistent underestimate of the size of the problem' [[14], p. 261]. Responses to the proposals raised in the present study, particularly those relating to mandatory measures, suggest that the employment of more demanding strategies further up the pyramid might be warranted. As Healy and Braithwaite emphasize, the ideal is not to *replace *persuasion with punishment, but to move up the pyramid when, and for as long as, mechanisms lower down fail to be effective. Many of the suggestions made by our expert panel indicate how this might be done.

### (1) Error Learning and Mandatory Disclosure

At the time of the QAHC study, it was not compulsory for hospitals and doctors to register AEs and routinely provide feedback to facilitate error learning. This means that the most important vehicle for improving quality and reducing patient risk was not compulsory. While open disclosure is mostly implemented in public hospitals now, the opportunity for error leaning is almost certainly under-utilised in some hospitals. Legislation might ensure the universality of this critical reform, given sufficient data processing mechanisms to enable policing. In Denmark mandatory reporting and comprehensive protection for reporting doctors has resulted in a high reporting rate with 50% of reports coming from doctors. This is no higher than 10% at the best sites in Australia and as low as 1% in some. The adverse events register in Australia could be linked to individual doctors and medical teams where appropriate, and suitable threshold levels installed that sequentially trigger information feedback for the purposes of review, followed by more active intervention. Despite this, nine years after publication of the QAHC study the chairman of the ACSQHC noted that 'we have insufficient accurate data for fully appreciating the current size of the multiple causes of this problem ... we need the data from multiple sources, including incident monitoring systems, routine administration data sources and the use of screening tools to practically identify areas that may cause harm' [[15], p. 5]. The mandatory reporting of AEs rated high among our experts in terms of its potential effect.

The published research on 'high reliability organizations' suggests that it is wise to separate information-gathering and inquisitorial processes from punishment such as dis-accreditation. Adverse events are unlikely to be reported if there is a financial incentive to hide the AE. For this reason legislative protection of doctors from the financial outcome of litigation is a reasonable prerequisite for a comprehensive, on-going process of error learning. The consequences for a doctor associated with an AE should be based upon medical criteria and uncoupled from the social mechanism for compensating patients, except where damage occurs due to negligence. In brief, 'the challenge for health care is to shift from a blame culture to a learning culture, in order to learn from adverse events' [[13], S57], as has occurred, for instance, in the aviation industry [16]. This was the view of our respondents, who gave a high effect rating to the proposal that doctors and hospitals should receive immunity against litigation relating to adverse events, and compensation to an injured party or parties should be paid for from a subsidised government or privately run claims-fund where compensation is not contingent upon blame. In the context of quality assurance activities the States and Territories have all now implemented measures to provide this protection.

### (2) Hospital Accreditation and Audit

For decades health professionals have believed that a significant number of small hospitals are dangerous. However, with full knowledge of the QAHC results, hospital accreditation remains voluntary in all States except Victoria. Although most public and private hospitals undergo formal accreditation procedures, the danger of self-selection remains. Low quality hospitals will not opt for accreditation and poorly qualified doctors will seek out these hospitals. Multiple accreditation teams could have the power to randomly inspect all hospitals or units within hospitals and (in the most extreme cases) close those judged to be dangerous – as occurs with restaurants with sub-standard hygiene. The proposal that universal accreditation should be mandated was rated 'high' by our experts in terms of its potential effect (P2.1).

In a written response to our survey, the Victorian Department of Human Services expressed the view that formal accreditation should occur on pre-arranged dates 'as this provides value in allowing hospitals to independently check, maintain, improve their systems prior to accreditation'. Another respondent thought the proposal unfeasible because 'hospitals take up to 12 months to self-evaluate'. Of course, non-random accreditation also allows hospitals 'to independently check, maintain, improve their systems prior to accreditation'. But the problem with accreditation on pre-arranged dates is that it may give an atypical picture of a hospital during the much longer non-review period. The time-consuming nature of the review process should not be underestimated but neither should the human cost of sub-standard hospitals. The Victorian DHS agrees that 'follow-up review and spot checks should be carried out on random dates'.

It is unclear whether or not present accreditation is sufficiently rigorous to reduce preventable adverse events significantly. There appears little reason why the accreditation process should not itself be reviewed to ensure that credentialed hospitals satisfy rigorous safety standards in their facilities and procedures. The proposal that there should be a review of the criteria for achieving accreditation, and that the criteria should be expanded to include more stringent procedures relating to safety (P 2.2), received a high impact rating from our experts.

To date, the majority of the reforms contemplated in government-commissioned reports represent process measures of success. However, their objective is to reduce adverse events and for this reason medical record analysis of the form conducted by the QAHC study should arguably be an on-going feature of the system. The QAHC study was relatively expensive, but these costs are small compared to the importance of the surveillance, the costs, the morbidity and the deaths averted. The proposal that the audit procedures used in the QAHC study should be introduced as a permanent feature of the public and private hospital systems, with mandatory auditing of identified high-risk hospitals, and random auditing of the remainder, was also judged favourably by our panel.

In 2005 the new Australian Commission on Safety and Quality in Health Care commissioned an advisory group to examine what data might be used to create a national dataset. The advisory group considered whether the QAHC study might be repeated, but concluded 'that the major difficulty of achieving consistent and reproducible definitions of 'adverse event' and 'preventable adverse event' would seriously hinder accurate comparisons of any new study with those of the past' [[17], p. S40]. However, it seems seriously misguided to allow such semantic obstacles to stand in the way of an important study which could produce independently important data. Any new study must be explicit about the definitions used, so that it is clear where comparisons are valid, where they are not, and where they are doubtful. But as Wilson and Van Der Weyden note, 'the absence of recent system-wide data on patient safety seriously hinders our ability to manage the problem and make improvements. Its absence makes a mockery of the tenets of continuous quality improvement' [[11], p. 260].

This is not to deny that there may be cheaper ways of gaining the same information than repeating the ACSQHC. For example, valuable information on adverse event rates can be obtained from routinely collected admissions data [5,18,19], although this method has its inherent limitations – e.g. adverse events that only manifest after discharge are likely to be missed.

### (3) Hospital Information Systems

Patient notes are still transferred within hospitals using 19th Century clipboards. It is known that this commonly causes potentially lethal errors. The mandated use of (long available) electronic forms of transmission could alert staff to the risk of inappropriate procedures, the administration of conflicting drugs or the failure to administer a drug. Likewise X-ray films are sometimes misplaced or lost. The consequences may again be lethal. Legislation could mandate the use of digital technology (with appropriate back-up systems and staff training) to ensure immediate access to results. New wireless technologies make it possible for roving staff – doctors and other professionals – to have constant access to text and basic technical data. There is no reason why much of the health system should have missed the IT revolution that has transformed other parts of the community. In relation to the size of the AE problem, the cost of implementing 21st Century information technology throughout the health system is likely to be small relative to the human and financial cost of AEs averted. Making it a condition of accreditation that 'all hospitals ... should have an appropriate internal information system for recording patient history, treatment (including drugs), digitized radiological imaging, pre- and post-discharge requirements' (P 3.1) was rated 'high' by our experts in terms of its potential effect.

### (4) Out of Hospital Information

A persuasive argument can be made that the public has a right to information relating to the performance of hospitals and individual doctors, provided 'that it is of high quality and able to be benchmarked in a valid way' [[20], p. S50]. There can be little doubt that, if consulted, the public would overwhelmingly endorse the need for this information. Additionally, the provision of information is an effective method for effecting change and it is likely that the pace of reform would be accelerated if the public was aware of the safety record of various health care providers. One argument against this is that the provision of information might result in a loss of confidence in hospitals and doctors. However, this fear seems to be unfounded [14]. The argument that the public should be kept in ignorance to engender unjustified confidence is, at best, dubious, and if this ignorance allows an inadequate policy response then it is additionally dangerous. In some states of the USA, and most notably New York, severity adjusted mortality rates are available for every hospital and for every doctor. This has not resulted in a significant change in the pattern for public demand but it has galvanised doctors and hospital staff to successfully review and upgrade their procedures [21]. League tables have recently been introduced in England to allow doctors and patients to evaluate the performance of particular hospitals [22]. From late 2004 the performance of individual surgeons will probably be available [23]. The impact of these measures can be expected to depend, inter alia, on public education.

As noted, our respondents were circumspect on the question of public access to the safety record of hospitals and providers of medical care (P 4.2), and thought it would take several years before any measures along these lines would have a major impact. Several of those surveyed indicated a particularly long timeframe – ten years or more – one suggesting that data regarding risk-adjusted mortality and adverse event by cause are slow to identify problems, both requiring more than 7 years to gain statistical significance. As noted earlier, the rather negative response to this proposal might, in part, be attributable to a desire to keep problems and solutions 'in-house' rather than tarnish professional reputations through publicity. However, it is hard to reconcile this with the later support for whistle blowing among our panel, and a more likely explanation is a belief that the public is ill-equipped to deal appropriately with the information. For example, as the Victorian Department of Human Services commented: 'There is not a sufficient level of sophistication or understanding of risk-adjusted mortality and adverse event by cause to make the information available to the public'. Arguably, however, this indicates the need for simple presentation of data, the provision of explanatory notes and public education. There is little reason to believe the Australian public is less able to appreciate this type of information than the UK and the US public. The important lesson from the latter experience is that publication of this data has not resulted in a negative response from the public but appears to have provided motivation for professional self-improvement.

More generally, access to data relating to health system performance, other than the material routinely published by government, is very difficult to obtain. As an example, access to Australia-wide, de-identified public hospital records requires the separate consent of all States and Territories as well as the co-operation of the Commonwealth Department of Health or AIHW (Australian Institute of Health and Welfare). Data linkage to determine the consequences of different treatment patterns – who lives and who dies – is so difficult that the research is effectively proscribed for most researchers.

### (5) Other Hospital Regulation

There is no regulation that links on-site expertise and the complexity or riskiness of the procedures that may be undertaken in a hospital. For example, it is possible for a hospital to permit significant surgery but have no on-site medical practitioner post-operatively. It was not until 2003 that the ACSQHC released a paper considering issues of staff rostering, skill mix, staff numbers, staff supervision and team functioning [7]. While endorsing the AMA (voluntary) code of practice [24], it comments – almost 8 years after the QAHC study – that 'responsibility for improving the management of staffing variables cannot [i.e. should not but still is being] left to individuals. It is a governance responsibility...' [[25], p. ii]. The proposal that regulation should require a defined minimum complement of qualified staff in situ (or in close proximity) following defined procedures in all public and private hospitals was judged by our experts to have a potentially high effect upon the reduction of AEs.

The proposal that all hospitals should have in place a risk management system that ensures personnel can initiate action to prevent and/or reduce the impact of risks, backed up by whistle-blower procedures that guarantee anonymity and/or protection (P. 5.2), received a 'high' effectiveness rating from our respondents, and in fact now exists in many hospitals. In general, the potential role of staff in adopting 'affirmative action' to reduce AEs was viewed very positively. This included support for the idea that all hospital staff should be trained in risk management so that staff assume 'ownership' of safety and quality issues (P 5.4).

### (6) Doctors

Patterns of private practice are already subject to scrutiny in Australia. But the chief purpose is to detect medical fraud. Legislation could require the examination of practices to detect those that deviate significantly from evidence-based guidelines constructed by the relevant Royal Colleges. When there is a known relationship between the small number of procedures carried out by a doctor and negative outcomes, as occurs with some types of surgery, critical annual procedure rates may be established that trigger the provision of information to the doctor, the mandatory review of the practice and finally, in the most extreme cases, the dis-accreditation of the doctor for the conduct of these procedures, perhaps contingent upon re-training. While it is true that some doctors take on the hard cases, partial standardisation for case complexity is possible, and this would obviously be taken into account by those conducting a review. Along these lines, a detailed national standard for credentialing and defining the scope of clinical practice has been produced by the ACSQHC [26].

The single proposal judged by our panel to have highest potential effect concerned the supervision and support of junior doctors (P 6.4). This was judged to be implementable within nine months and likely to have a major impact upon AEs within another seven months. Similarly, the proposal that all medical students who become interns should be 'credentialed' before being allowed to undertake any unsupervised procedures was rated high (P 6.3). While flawed systems and procedures are clearly implicated in the occurrence of AEs, these latter results suggest that human error plays an important role in the occurrence of AEs.

### (7) System Level Reform

Financial incentives are one of the most effective, non-coercive ways of achieving desired outcomes and numerous economic studies have demonstrated their effectiveness. In Australia there has been limited use of this powerful instrument and the financing of medical services has generally been perceived as a reward for providers doing what they select to do rather than as an opportunity for influencing what is done. This is an important missed opportunity. (As one of our respondents pointed out, retrospective payment for safety-related practices will probably reward well-endowed hospitals, providing one reason for using prospective payment.) Financial incentives are non-coercive and avoid the head-to-head conflict between 'clinical autonomy' and the 'patient's right to evidence-based medicine' that may accompany direct regulation. The proposal that higher payments should be made throughout the public and private system for practices that are known to improve safety received a high potential effect rating, but with implementation and impact times stretching into years rather than months [27-29].

In its Fourth Report, the ACSQHC notes a number of State initiatives aimed at the reduction of AEs [15]. But there is no reference to national legislative action to enforce safety. As Healy and Braithwaite note: 'there are no national published measures of adverse events, despite the beginnings of state-based monitoring of sentinel events. Without some form of standardised reporting, there is no way to benchmark performance and to systematically trace progress' [[13], p. S57]. Indeed, there is some evidence that the ACSQHC was itself frustrated with the scale of the national effort. For example, in a recent *Medical Journal of Australia *article its Chairman comments: 'one might assume that systematic improvements within the health system are either happening or, at the least, well advanced. Regrettably, improvements are still patchy. The greatest challenge for all remains how to achieve universal and systemic changes to the health system within a federated system' [[20], p. S49].

Among our panel there was support for such leadership – for example, the establishment of a National Centre for The Development of Clinical Guidelines and Clinical Pathways, which would promote evidence-based practice, disseminate evidence-based clinical guidelines, and prepare model clinical pathways to assist hospitals plan and organise care.

### The Methodology

Our study was, in part, illustrative of what might have, but did not, happen following publication of the QAHCS. Ideally this would have involved a much larger-scale study, and have canvassed suggestions from any individual or group in a position to make useful suggestions.

The reported research was conducted with a limited budget and with no 'official endorsement' – e.g. from the Australian Medical Association. This narrowed the number and range of those who could be surveyed. Unsurprisingly, the response rate was low (but typical for 'cold' mail questionnaires). The options for reform suggested were general, not detailed, and the time-lines nominated were subjective. The scope and detail of the suggestions are necessarily more limited than would have been outlined with a large, official survey. However, the conclusion that any of these factors invalidates the research or undermines its credibility would miss the point of the study.

The study was based on the belief that a single idea from a single person, irrespective of their authority, may contribute to a reduction in unnecessary deaths. The minimum acceptable sample size is '1' if the suggestions obtained are valuable. The antithesis of this approach is the view that action should be delayed until 'due process' has been followed, consisting of the agreement or consensus of appropriate authorities. The cost of such delay, however, is loss of life and permanent injury.

Our survey was akin to an organised 'brain storming' exercise with feedback. Suggestions are a starting point, not an endpoint for policy reform and development. Likewise, the nominated time-lines are indicative of a view among some well-informed commentators that action following the QAHC study could have been significantly swifter. They do not purport to reflect objective data.

While the low response rate is of limited methodological relevance it was disappointing. We expected that, given the gravity of the subject, we might have obtained a higher rate. Informal feedback suggested one likely reason. The authors, being social scientists, would be perceived as having little authority, credibility or legitimate role in the field of service delivery and safety; that their research should have been limited to cost-benefit analysis. This response may be indicative of a 'closed shop' culture: the safety of our health services is a matter for accredited medical experts operating from within approved institutions with approved channels for effecting reform – a suggestion also made by others [[13], p. S56]. If correct, this attitude, in conjunction with inadequate governance of quality, might go some way towards explaining the sluggish response in the field to the QAHC report and, of course, the large number of unnecessary deaths caused by this. It suggests the need for a governance that seeks and is more responsive to a broadening of the input and approach to such a multi-faceted problem as AEs, and in which the magnitude of the problem, as documented by the QAHCS, would warrant government involvement at the highest level.

The appropriate test of the validity of a method, in this area, is whether or not it elicits useful suggestions which have not, to date, been canvassed or carefully examined. Our incomplete reading of the literature suggests that our minimalist research effort indeed identified options with the potential for saving lives – and did so quickly and at little expense. If correct, this reveals a significant deficiency in the methods used over the past fifteen years for governance of quality and safety in Australia's hospital system.

## Conclusion

Relative to the size of the problem, the response to the QAHC study was very surprising, to say the least. The study authoritatively documented what was arguably the most dramatic and serious problem ever found in the health system – and possibly the nation as a whole. Annual deaths from AEs were initially estimated to be equivalent to 13 jumbo jet crashes each year, each resulting in 350 deaths: events that would surely have galvanized rapid and decisive action. The lack of effective action that followed publication of the QAHC study revealed a fundamental failure of governance by both the State and the Commonwealth governments and an apparent lack of willingness to respond appropriately at both the bureaucratic and political levels.

In terms of the magnitude of death and injury involved, an analogy with a war casualty rate is not unjustified. In the face of ongoing casualties, decision makers in war time must exercise judgement and take responsibility for a rapid response. With an estimated 50 Australians dying daily and another 140 sustaining permanent injury, at the time of the QAHC study, the appropriate criteria for immediate action should have been 'likely cause' and 'likely solution' not 'confirmed, demonstrated cause' or 'solution based on professional consensus'. This type of decision making clearly did not occur in Australia following the release of the QAHC study.

Historically, safety and quality control of the health system has relied on internal rather than external monitoring: 'the state generally has left the regulation of health care performance to the medical profession' [[13], p. S56]. However, the reliance on voluntary regulation has seen public confidence in the health system shaken, particularly in the wake of media reports highlighting some very upsetting healthcare 'scandals' [30]. The result has seen the emergence of new regulatory bodies, the production of numerous reports, and the expenditure of millions of dollars, but the rate of change still appears to be slow. As the Chairman of the ACSQHC commented: 'the new Commission [on Safety and Quality in Health Care] must not only recommend reforms to ministers, but be able to push jurisdictions to move at a faster pace than in the past' [[20], p. S49]. The new commission has no ability to affect jurisdictions despite recommendations that it should have such powers.

The ACSQHC faced numerous obstacles during its six-year tenure [20] and worked hard to improve the safety and quality of health care in Australian hospitals. Its achievements should not be underestimated. Despite this, systematic change has been slow in coming. In an Editorial in the *Medical Journal of Australia *in 2005, a member of the Council asked, 'Ten years on can we confidently state that healthcare is safer for patients?' and answered forthrightly, 'There is insufficient information at a state or national level to determine whether any or all of the efforts over the past 10 years have increased safety in our hospitals' [11]. The purpose of the present paper was not to publish a blueprint for reform or to claim that our suggestions are authoritative but to demonstrate the possibility of a more rapid and vigorous response to the challenge of AEs and to raise some practical suggestions, which, we hope, may still help to improve the safety and quality of health care in Australia.

## Competing interests

The authors declare that they have no competing interests.

## Authors' contributions

Both authors contributed equally to this work.
